# Hereditary Creutzfeldt-Jakob Disease: A Case Presentation of a Rare Stroke Mimic

**DOI:** 10.7759/cureus.55559

**Published:** 2024-03-05

**Authors:** Rachel E Bridwell, Jessica A Barlow, Andrew R Jacobson, Angela Curell, Brit Long

**Affiliations:** 1 Emergency Medicine, Madigan Army Medical Center, Joint Base Lewis-McChord, USA; 2 Emergency Medicine, Kaiser Permanente, Roseville, USA; 3 Anesthesiology, Brooke Army Medical Center, San Antonio, USA; 4 Anesthesiology, University of Cincinnati Medical Center, Cincinnati, USA; 5 Emergency Medicine, Brooke Army Medical Center, Fort Sam Houston, USA

**Keywords:** prion diseases, transmissible spongiform encephalopathy, stroke mimic, hereditary, creutzfeldt jakob disease

## Abstract

Acute ischemic cerebrovascular accident (CVA) is a time-sensitive emergent diagnosis, requiring rapid diagnosis and consideration of thrombolytic administration. However, a myriad of cerebrovascular mimics creates a diagnostic challenge. A rare CVA mimic is Creutzfeldt-Jakob disease (CJD), a rapidly progressive fatal dementia due to protein misfolding. Magnetic resonance imaging (MRI) and neurology consultation for electroencephalogram (EEG) and specialized cerebrospinal fluid (CSF) studies are diagnostic while the patient is alive. All forms are fatal within months, and diagnosis can be confirmed on postmortem brain testing. While incredibly uncommon, emergency clinicians should consider this diagnosis in the proper patient to advocate for specialized CSF testing and potential palliative care consultation.

## Introduction

With 79,500 people visiting the emergency department (ED) annually for new or recurrent episodes, cerebrovascular accident (CVA) represents a major and temporally sensitive diagnosis in the ED [1]. Current American Heart Association guidelines recommend the administration of thrombolytics within the 0- to 4.5-hour window if all criteria are met, further emphasizing the importance of timely stroke identification [2]. While this highlights the importance of focused screening for CVAs in the ED, CVA mimics present a clinically challenging conundrum for emergency providers, as thrombolytic administration is not without risk. One rare mimic is Creutzfeldt-Jakob disease (CJD), a rapidly progressive fatal dementia secondary to a protein-mediated prion disease associated with misfolded proteins in the central nervous system [3,4]. The authors present a case involving a 73-year-old male who presented with diplopia and ataxia, was admitted for CVA evaluation, found to have CJD, and died seven days following admission.

## Case presentation

**Figure 1:**
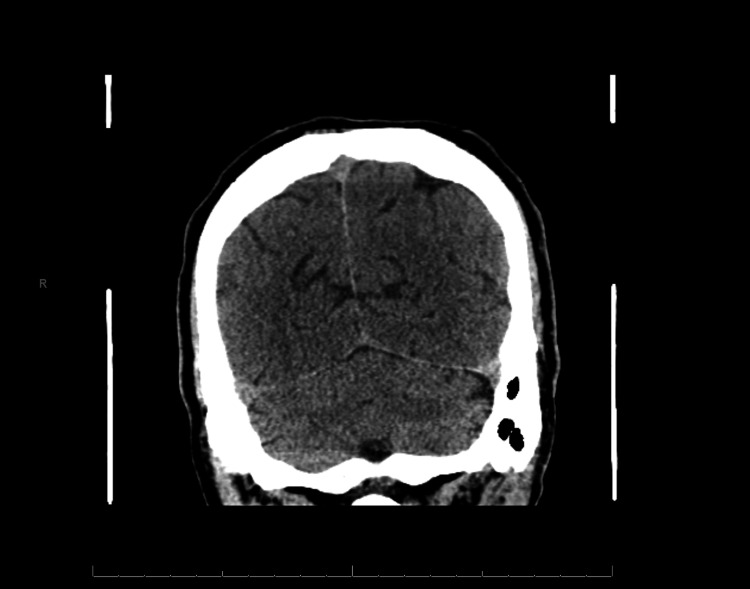
Non-contrasted head computed tomography demonstrating only senescent changes with cortical atrophy appropriate for age.

A 73-year-old male with a history of medication-controlled benign prostatic hypertrophy and gastroesophageal reflux disease presented to a community ED complaining of 10 days of diplopia and ataxia. Ten days prior, the patient had visited a chiropractic clinic for neck pain, received advice to use a massage gun, and subsequently developed intermittent diplopia on lateral gaze in both directions, along with blurry vision and ataxia, without any provoking or relieving triggers. He denied any changes in medication, new supplements, or recreational drug use, as well as any recent trauma. On examination, his vitals were an oral temperature of 99.4 °F, blood pressure of 122/73 mmHg, heart rate of 68 beats per minute, and a respiratory rate of 16 breaths per minute with a saturation of 98% on room air. His physical exam was without nystagmus with normal extraocular movements notable only for an ataxic gait. His laboratory evaluation, including a complete blood count, comprehensive metabolic panel, liver function tests, urinalysis, prothrombin time, and international normalized ratio, was otherwise unremarkable. A computed tomography (CT) of the head without contrast and CT angiography of the head and neck showed no evidence of intracranial hemorrhage or large vessel occlusion (Figure 1).

**Figure 2:**
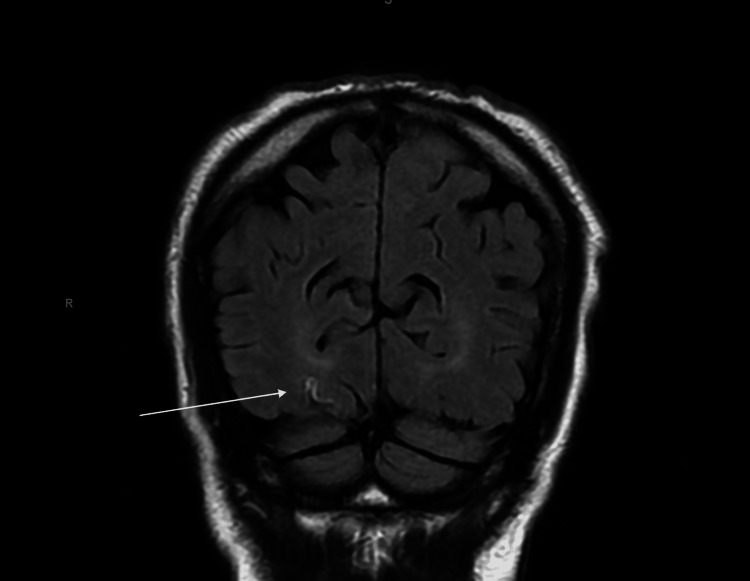
Magnetic resonance imaging without contrast of the brain demonstrated subtle areas of cortically based reduced effusion along the medial aspect of the right occipital lobe, consistent with CJD. White arrow: cortical ribboning sign showing reduced effusion. CJD, Creutzfeldt-Jakob disease

A magnetic resonance imaging (MRI) obtained in the ED of the brain demonstrated restricted diffusion in the right occipital and posterior right temporal lobes, raising concerns for a CVA as well as CJD, as communicated by the radiologist in real-time (Figure 2).

The patient was admitted to the hospital with neurology consultation, which favored the diagnosis of posterior ischemic CVA. On further history, he denied recent ingestion of meat, corneal transplants, neurosurgery, or foreign travel. A lumbar puncture performed by neurology to evaluate for CJD demonstrated mildly elevated protein with a positive real-time quaking-induced conversion amplification (RT-QuIC) for prions, elevated T-tau protein, and an elevated 14-3-3 gamma level (Table 1).

**Table 1:** Cerebrospinal fluid studies from the patient on hospital day 1.

Cerebrospinal fluid marker	Finding	Reference range
Glucose	52 mg/dL	50-80 mg/dL
Protein	61 mg/dL	15-60 mg/dL
Red blood cells	0 cells/uL	0-5 cells/uL
Positive real-time quaking-induced conversion amplification (RT-QuIC)	Positive	N/A
T-tau protein	3,675 pg/mL	0-1,149 pg/mL
14-3-3 gamma protein	25,925 AU/mL	<30 to 1,999 AU/mL

These cerebrospinal fluid (CSF) results solidified the diagnosis of hereditary CJD, although the patient denied any known family history, as he was adopted. A repeat MRI on hospital day 2 demonstrated the pulvinar sign, hyperintensities in the pulvinar thalamus on fluid-attenuated inversion recovery (FLAIR), and reduced effusion in the bilateral occipital lobes and posterior right temporal lobe, which correlated to the patient’s loss of visual acuity. An electroencephalogram (EEG) demonstrated focal slowing in the right temporal region. Although this can be seen in other neurologic conditions, when considered in combination with rapid symptom progression, CSF studies, and MRI, this was concerning for CJD. By hospital day 4, he developed abnormal upper and lower extremity movements with a complete loss of vision. At this point, he opted for an elective end-of-life program and was discharged home. When the local end-of-life program responded on the next business day, the patient was too mentally incapacitated by CJD to enroll, and he died 48 hours later.

## Discussion

Although rare, CJD is a subset of transmissible spongiform encephalopathies that can be sporadic, familial, iatrogenic, or infectious. The most well-known among them is bovine spongiform encephalopathy, which can be transmitted through contaminated meat consumption [5]. As a result, the peak incidence occurred in 2000 secondary to the mad cow disease epidemic. Since then, the global incidence has stabilized at 1-2 cases per million, though this varies by geographic location [4]. CJD is hypothesized to occur secondary to a mutation in the Prion Protein Gene (PRNP), causing the misfolding of Prion Protein C (PrP^c^) into the scrapie isoform of Prion Protein (PrP^Sc^). This accumulation in the CNS results in a progressive and rapidly fatal disease [6]. While less common than spontaneous CJD, hereditary CJD accounts for 10%-15% of all cases, with an autosomal dominant inheritance and high penetrance. However, often no family history is known, although genetic referrals can assess for PRNP mutations [7-9]. Unfortunately, all forms are fatal within five months [7].

The presentation can vary in age and is influenced by the area of the brain affected, as well as the particular form of transmissible spongiform encephalopathy in question. However, sporadic CJD classically presents with ataxia, involuntary myoclonic movements, and progressive dementia [10]. However, symptoms can include frontotemporal dementia, cerebellar ataxia, cognitive and sensory changes, sleep disorders, autonomic disturbances, and gait issues, depending on the mutation subtype and area of the brain that is impacted initially [10]. Emergency clinicians should obtain information concerning family history and history of organ transplant, neurosurgery instrumentation, blood product transfusion, and foreign travel, which may highlight any acquired etiologies [5]. A careful neurologic examination should include visual acuity, looking for any new decreases, and a trial of ambulation, which may highlight ataxia and/or sensory changes [11,12]. These patients may also demonstrate cognitive deficits and uninhibited behavior, especially in frontotemporal CJD [4].

Emergency clinicians must consider other conditions on the differential, including meningitis, brain abscess, sepsis, trauma, and toxicological etiologies, and thus, the ED evaluation may include laboratory assessment, head CT, lumbar puncture, and MRI if available. There are no current U.S. Food and Drug Administration-approved serum tests for CJD [13]. While CT can be evaluated for other conditions, MRI has utility in viewing the basal ganglia and cerebral cortex, which can demonstrate increased signal density [14]. FLAIR sequences may demonstrate irregularities in the putamen or caudate nucleus, which are 87% sensitive and 91% specific, although hyperintensities within the medial region of the occipital, parietal, and frontal lobes are common [14,15]. EEG may assist and demonstrate triphasic periodic complexes [11]. These support the diagnosis of CJD, although CSF studies have largely supplanted EEGs with increased specificity [11]. In this particular case, neurology assessed this patient to be most likely affected by hereditary CJD rather than sporadic CJD, based on his age at presentation, rapid occipitotemporal progression, and CSF testing. However, confirmation would require parenchymal staining and genetic testing postmortem.

With increasingly sensitive technology, CSF markers have aided in the diagnosis of individuals with a clinical picture and diffusion-weighted imaging (DWI). Both the 14-3-3 protein and RT-QuIC amplification have shown high diagnostic yield [16]. In a recent meta-analysis, T-tau was found to have low sensitivity, while RT-QuIC demonstrated higher sensitivity compared to the 14-3-3 protein [17]. Due to the rapidly fatal nature of this disease, confirmatory testing can be performed postmortem on brain parenchymal tissue [7,11]. While this is an incredibly rare disease, one previous case series outlines 30 cases that presented as ischemic CVA mimics [13]. Despite technological advances in MRI and DWI since the publication of these 30 cases, all patients were admitted with CVA consultation to neurology, necessitating additional inpatient investigation [13]. Due to the universally fatal nature of CJD, palliative care consultation should be sought as soon as the diagnosis is confirmed, and any family members should be offered a genetics referral.

## Conclusions

CVA is a frequent and time-sensitive ED diagnosis, although the clinical picture can be muddled by a variety of mimics. A rare but universally fatal mimic is CJD, which occurs in sporadic, familial, iatrogenic, or infectious variants. These patients can present with significant neurologic abnormalities and deficits. While CT is useful to rule out other etiologies, MRI, EEG, and CSF studies can help solidify the diagnosis. However, the diagnosis can be confirmed only with postmortem brain parenchymal staining. Unfortunately, this diagnosis is universally fatal within days to months. Emergency clinicians should consider rare CVA mimics, especially in those without acute intracranial hemorrhage and progressive neurologic symptoms.
